# The influence of globalization on medical regulation: a descriptive analysis of international medical graduates registered through alternative licensure routes in Ontario

**Published:** 2016-12-05

**Authors:** Wendy Yen, Kathryn Hodwitz, Niels Thakkar, Maria Athina (Tina) Martimianakis, Dan Faulkner

**Affiliations:** 1 The College of Physicians and Surgeons of Ontario; 2 The University of Toronto; 3 The Wilson Centre, Toronto

## Abstract

The increasing globalization of the medical profession has influenced health policy, health human resource planning, and medical regulation in Canada. Since the early 2000s, numerous policy initiatives have been created to facilitate the entry of international medical graduates (IMGs) into the Canadian workforce. In Ontario, the College of Physicians and Surgeons of Ontario (CPSO) developed alternative licensure routes to increase the ability of qualified IMGs to obtain licenses to practice. The current study provides demographic and descriptive information about the IMGs registered through the CPSO’s alternative licensure routes between 2000 and 2012. An analysis of the characteristics and career trajectories of all IMGs practicing in the province sheds light on broader globalization trends and raises questions about the future of health human resource planning in Canada. As the medical profession becomes increasingly globalized, health policy and regulation will continue to be influenced by trends in international migration, concerns about global health equity, and the shifting demographics of the Canadian physician workforce. Implications for future policy development in the complex landscape of medical education and practice are discussed.

## Introduction

Globalization is not a new phenomenon, but technology and policy development in the past few decades has spurred unprecedented increases in cross-border trade, investment, and migration.^1^ The increasing globalization of the medical profession has influenced health policy, health human resource planning, and medical regulation in Canada. Developing effective policies to address Canada’s health human resource needs has historically been complex but globalization trends such as the outsourcing of medical education and increased physician migration has introduced new challenges for policy makers, educators and regulators.^2^ This paper explores demographic trends in a subset of internationally trained medical graduates (IMGs) who enter the licensure process through alternative pathways developed by the College of Physician and Surgeons of Ontario (CPSO). In the process, we will also characterize the diverse routes to licensure of all IMGs in Ontario and highlight the broader intersections between globalization, health policy, and medical regulation.

Physician migration has grown in the twenty-first century, and Canada continues to be a primary recipient of IMGs.^3^,^4^ Nearly 25% of practicing physicians in the country are IMGs, with reliance varying by province.^5^ Ontario, the largest and most populous province in Canada, is central to the ebbs and flows of national and international migration, with 28% of its physician workforce having trained internationally.^6^ Canada has long relied on IMGs to fulfill health human resource needs, but in the last decade there has been more attention paid to facilitating the entry of these physicians into the Canadian workforce.^7^

In the early 2000s, physicians and policy-makers in Ontario predicted a significant physician shortage; tens of thousands of Ontarians were projected to be at risk of having poor access to physician services.^8^–^10^ Since then, a number of policy initiatives were implemented to increase integration of IMGs and to ameliorate predicted physician shortages.^8^–^10^ These policies included increased number of government funded residency positions for IMGs, increased enrolment in undergraduate medical education, increased flow of IMGs from other provinces, and targeted marketing and recruitment efforts.^9^,^10^ In Ontario, the CPSO created new policies for licensure and developed methods to ensure the competence of IMGs once registered.

As the medical regulator in Ontario, the CPSO issues certificates of registration (i.e., licenses) to physicians, and monitors and maintains standards of practice to ensure patient safety. The CPSO was a key stakeholder in the Task Force on Licensure of International Medical Graduates that provided recommendations for the entry of IMGs into Canada.^11^ In response to the predicted physician shortages, and in accordance with the Task Force recommendations, the CPSO developed a number of alternative licensure routes to increase the opportunities for qualified IMGs to obtain licenses to practice in Ontario.

**Figure 1:**
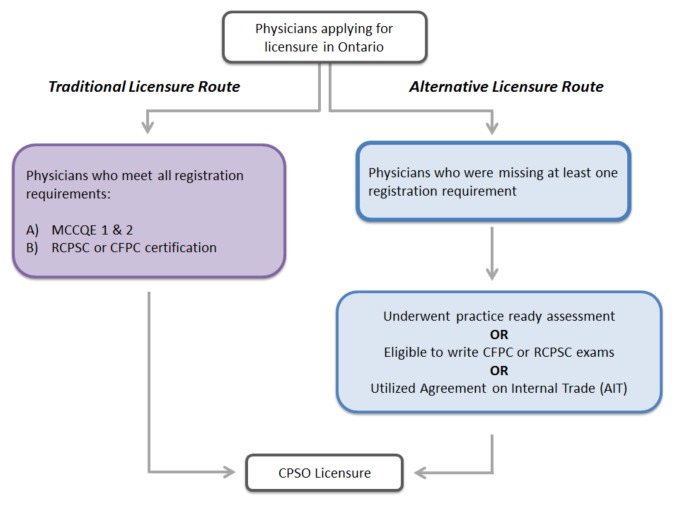
Traditional and alternative licensure routes for physicians in Ontario MCCQE – Medical Council of Canada Qualifying Examinations || RCPSC – Royal College of Physicians and Surgeons of Canada || CFPC – College of Family Physicians of Canada || Agreement on Internal Trade (AIT) – labour mobility legislation that allows free movement of physicians across provinces

In order to be eligible for a license in Ontario, physicians need to meet the registration requirements articulated in the Medicine Act of 1991. This includes successful completion of both the Medical Council of Canada Qualifying Examinations and one of the national certification examinations (i.e., the Royal College of Physicians and Surgeons of Canada (RCPSC) or the College of Family Physicians of Canada (CFPC)). We refer to this as the *traditional licensure route*, and it represents the route for the majority of physicians in the province. Beginning in 2000, *alternative licensure routes* were created for physician applicants who did not meet all the regulatory requirements but who met a series of alternative qualifications set out by the CPSO, primarily for those with recognized training and experience obtained internationally (see Figure 1). Practice ready assessment programs were implemented for IMGs and various policies were developed to recognize previous practice experience to meet eligibility requirements to write RCPSC and CFPC exams. Federal labour mobility legislation, the Agreement on Internal Trade (AIT),^12^ was also introduced that allows for increased migration of physicians across provinces.

Previous studies have examined IMGs who are practicing or training in Canada through other policy initiatives, most of whom completed a Canadian residency program and met the traditional licensure requirements in Ontario.^13^–^15^ The current study provides demographic trends and descriptive information about a unique group of IMGS: those who registered through the CPSO’s alternative licensure routes. One of the key criticisms of the current literature on IMGs is the tendency to treat all IMGs as a single homogenous group despite key differences in training, credentialing, and practice experience.^16^ Our analysis of medical regulatory data captured at the CPSO is the first to examine specific subgroups of IMGs and aims to strengthen the IMG literature base by exploring the demographic characteristics and career trajectories of all IMGs.

The purpose of this study is to describe the physician population in Ontario and highlight how globalization has influenced health policy, medical regulation, and trends in the physician workforce. Monitoring and reporting on the training, practice, and demographic characteristics of various groups of IMGs is an important first step in understanding how globalization and related policy initiatives have shaped the physician population over the last 15 years. The unintended consequences of these policies will be explored and new policy initiatives that have been developed to respond to trends in globalization will be described. As medical education and practice continues to globalize, future policy development will be impacted by steady increases in international migration, concerns about global health equity, and the changing demographics of the physician workforce. Given the constantly evolving nature of this area of research, it behooves researchers and policy makers to continue to mine physician data to inform future policy development at the level of the medical regulator and the health care system.

## Methods

### Sample

This was a retrospective cohort study using the CPSO Registry database. The analytical sample used was a subset of physicians registered with the CPSO between 2000 and 2012. The CPSO membership includes both IMGs, defined as physicians who received an undergraduate medical degree outside of Canada and the United States, and DMGs (domestic medical graduates), defined as physicians who received an undergraduate medical degree in Canada or the United States. Both Canadian- and American-trained physicians were considered domestic graduates because of the joint accreditation between Canadian and American medical schools.^17^

All physicians can broadly be dichotomized into two categories based on their credentials at the time of licensure: Traditionally Licensed Physicians (TLP) and Alternatively Licensed Physicians (ALP) (see Figure 1). In the twelve-year analytical time period, the majority of ALPs were IMGs. Understanding the characteristics of these IMGs (ALP-IMGs) was a primary focus of this study. Therefore, we examined the demographic and practice characteristics of these physicians in comparison to IMGs who were registered through the traditional licensure route (TLP-IMGs). TLP-DMGs were included for reference. We also further examined the ALP-IMGs by dividing them into three sub-groups based on migration patterns and eligibility criteria to practice in Ontario. Each of the study groups are described in Table 1.

All physicians in the analysis held an active licence in Ontario as of March, 2015. Traditionally licensed physicians with a postgraduate training certificate or a restricted certificate as of March, 2015 were excluded from analyses. Alternatively licensed DMGs were also excluded as they were not the focus of the present study.

### Variables

The variables used in this study to describe the demographic and practice characteristics of physicians in Ontario were age at time of registration, sex, location of undergraduate medical school (grouped by geographical region and Human Development Index (HDI^18^), current specialty, and practice location (as of March, 2015).

Geographic regions were included in this study to provide a more nuanced description of the training location and career path of IMGs. The Human Development Index is a measure of the development of a country based on life expectancy, education, and standard of living as it relates to gross national income per capita. This measure has been used previously to classify and compare IMGs.^19^ HDI provides a rank order of each country and classifies countries into four strata based on their ranking: very high, high, medium, and low human development.^18^

Specialty was grouped into seven practice foci: Family Medicine, Medical Specialties, Surgical Specialties, Diagnostic Specialties, Psychiatry, Pediatrics, and Anesthesiology & Critical Care. This allowed for practice characteristics to be divided into clinically meaningful groups while maintaining anonymity of individual physicians. Rurality was used to examine differences in physicians’ current practice location and was based on primary practice address. Using 2008 Rurality Index of Ontario (RIO) codes,^20^ physicians were grouped into one of three classifications: urban, suburban, or rural.^21^ All physicians held an active licence in Ontario as of March, 2015; missing practice location data was due to physicians having a primary practice location outside of Ontario.

### Data Extraction and Analysis

Ethical approval to undertake this study was obtained from the University of Toronto Research Ethics Board. Data were obtained from the CPSO Registry database and from physician-reported registration files. Descriptive statistics were generated for each of the variables using SPSS.

## Results

The demographic and practice characteristics of the physicians included in this study are displayed in Table 2. Cell sizes of less than six have been supressed for anonymity.

### 1: Routes to licensure

A total of 11,250 physicians were included in the study. Of the 10,595 traditionally licensed physicians (TLPs), 73% were domestically trained (DMGs) and 27% were internationally trained (IMGs). The 655 alternatively licensed IMGs (ALP-IMGs) accounted for 6% of the total study population. Twenty percent (20%) of the ALP-IMGs migrated to Ontario from another province and obtained licensure either through labour mobility legislation (AIT) or by becoming eligible for the CFPC licensing exams due to prior experience (ALP-Canadian Practice Experience IMGs). The ALP-IMGs who were considered practice ready either through assessment or completion of post-graduate training in a RCPSC-approved jurisdiction (ALP-Practice Ready IMGs) comprised 22% of the ALP group and those who completed North American postgraduate training comprised 58% of ALPs (ALP-North American Trained IMGs).

### 2: Demographic Characteristics

On average, ALP-IMGs were nine years older than TLPs at time of registration (40 vs. 31 years). TLP-DMGs were the youngest (29 years), followed by TLP-IMGs (37 years), and ALP-IMGs (40 years). Within the ALP-IMGs, the Canadian Practice Experience IMGs were the oldest sub-group at 44 years at time of registration.

There were proportionally more male ALP-IMGs than male TLPs (59% vs. 49%). TLP-DMGs had the lowest proportion of males (48%), followed by TLP-IMGs (54%) and then by ALP-IMGs (59%). The ALP-Canadian Practice Experience IMGs had the highest proportion of males (73%).

### 3: Education and Training

The regions of medical school for ALP-IMGs were diverse: those who completed their undergraduate medical degree in the Indian Subcontinent made up the largest proportion (28%), followed by those who trained in the Middle East (17%), the Caribbean (13%), and Eastern Europe (12%). The top five countries of medical school for ALP-IMGs were India, Pakistan, Egypt, South Africa and Iran; the top five countries for TLP-IMGs are almost the same: India, Pakistan, Egypt, Ireland and Iran. Nearly 50% of both TLP-IMGs and ALP-IMGs attended medical school in the Indian Subcontinent and the Middle East. There were slightly more IMGs from Western and Eastern Europe in the TLP group compared to the ALP-IMG group (24% vs. 18%).

More TLP-IMGs were trained in countries with a very high HDI compared to ALP-IMGs (23% vs. 13%). The top five countries of medical school for TLP-IMGs from very high HDI countries were Ireland, United Kingdom, Saudi Arabia, Australia and Poland, accounting for nearly 80% of the very high HDI group. Despite the elevated number of TLP-IMGs from very high HDI countries, about half of all IMGs are from countries with a low or medium HDI. While only a small proportion of all IMGs are from very high HDI countries, the majority of these IMGs registered through the traditional licensure route compared to the alternative routes.

### 4: Practice Characteristics

#### Specialty

The practice focus of physicians differed slightly across the study groups. TLP-IMGs had a higher proportion of Family Medicine physicians than TLP-DMGs (57% vs. 47%). There were proportionally fewer ALP-IMGs practicing in Family Medicine (44%) than either TLP-IMGs or -DMGs. The sub-group with highest proportion of physicians specializing in Family Medicine (67%) was the ALP-Canadian Practice Experience IMGs while the ALP-Practice Ready IMGs had the highest proportion of surgical specialists (21%) and physicians practicing in anesthesiology and critical care (15%). ALP-North American Trained IMGs had the highest proportion of medical specialists (21%) and psychiatrists (11%).

#### Practice Location

There were no substantial differences in rurality of practice location across groups; the majority of all physicians practiced in urban centers. TLPs had a relatively higher disparity between urban and rural practices (85% vs. 4%) compared to ALP-IMGs (78% vs. 4%). A higher proportion of ALP-IMGs practiced in suburban areas compared to TLPs (18% vs. 11%). The ALP-Practice Ready IMGs had the highest proportion of suburban practice (24%) and the lowest proportion of urban practice (72%). Approximately 5% of all physicians in this study practiced in northern Ontario. Thirty-three percent (33%) of TLPs in northern Ontario had rural practices, whereas 50% of ALP-IMGs in northern Ontario practiced rurally.

## Discussion

To our knowledge, this is the first study that has examined the demographic and practice characteristics of all IMGs practicing in Ontario. By reporting on the IMGs registered between 2000 and 2012, we hope to shed light on the diverse routes to licensure of IMGs in Ontario and highlight some of the intersections between globalization, health policy, and medical regulation.

### Routes to Licensure

In Ontario, nearly 3,500 IMGs entered the healthcare system in a 12-year timeframe, with 655 IMGs accessing CPSO alternative licensure routes (ALP-IMGs). Of the ALP-IMGs, the majority were ALP-North American Trained IMGs, who entered practice in Ontario by completing postgraduate training in North America. This includes IMGs who trained in the US as well as those who trained in Canada but had not yet written the certification exams at the time of requesting licensure. The second largest subgroup was the ALP-Practice Ready IMGs, who were deemed ready to practice by completing postgraduate training in a jurisdiction approved by the RCPSC (e.g. the United Kingdom, Australia, New Zealand, Hong Kong, South Africa) or by undergoing a practice ready assessment in Ontario. Practice-ready assessments were designed for internationally trained medical specialists, most of whom had foreign practice experience prior to entering Ontario.

The third sub-group, the ALP-Canadian Practice Experience IMGs who migrated to Ontario from another Canadian province, comprised 20% of ALP-IMGs. This is unsurprising given that Ontario has long had the highest net gain of physicians nationally, while other provinces such as Newfoundland, Saskatchewan, and Manitoba regularly have net losses of practicing physicians.^22^ These three provinces have historically recruited internationally trained physicians to address their physician shortages; however, upon completion of return-of-service agreements to practice in underserviced areas, many IMGs move to urban regions or to other provinces, particularly Ontario, British Columbia, and Alberta.^23^

Multiple studies have demonstrated that smaller provinces provide an entry point for IMGs to practice elsewhere in Canada.^24^–^26^ In some of these jurisdictions, IMGs were permitted to practice without Canadian post-graduate training and/or the necessary certification exams. In an effort to standardize licensure requirements, the Federation of Medical Regulatory Authorities of Canada has facilitated the development of common medical licensure approaches for all Canadian jurisdictions.^27^ In addition, there is a pan-Canadian initiative under the Medical Council of Canada to develop and implement a common examination for IMGs seeking a first year Canadian postgraduate position as well as common standards for the delivery of practice ready assessments across provinces.^28^ This will not limit mobility between provinces, but will serve to standardize the evaluation of IMGs’ qualifications and the assessment of competence. As physician migration continues to increase both nationally and internationally, policies that facilitate access to licensure while ensuring consistent, high quality patient care are imperative.

### Demographic Characteristics

All IMG groups were older and had proportionally more males than the TLP-DMG group Earlier comparisons of IMGs and DMGs in Canada have also found these phenomena.^13^,^29^,^30^ The noted age difference can be explained, at least in part, by the fact that many IMGs have completed training or have practiced (in their home countries or in Canada) prior to registering in Ontario. ALP-IMGs, specifically, enter Ontario later in their careers than TLP-IMGs. On average, ALP-IMGs had almost five years of training and over four years of practice outside of Canada prior to registering in Ontario. Those who migrated from another Canadian province, the ALP-Canadian Practice Experience IMGs, were the oldest and had 12 years of practice experience prior to registering in Ontario. Physicians who utilized labour mobility legislation (AIT) who were, on average, 45 years of age at time of registration. The ALP-Practice Ready IMGs and ALP-North American Trained IMGs were slightly younger, and had nine and five years of prior experience, respectively.

These findings highlight the diversity of experience of IMGs and the often circuitous routes to licensure taken by these physicians. They also suggest that IMGs may have shorter careers in Ontario than DMGs since they are older when they enter the workforce. Given that increasing access to licensure for IMGs is intended to contribute to overall physician supply, policies to recruit younger IMGs and reduce potential barriers to entry would allow IMGs to have longer careers in Ontario and have a greater impact on the healthcare system.^13^

### Education and Training

The diverse paths of IMGs are evident in the wide range of source countries of IMGs now practicing in Ontario. The majority of IMGs in the present study trained in the Indian Subcontinent, the Middle East, Europe, and the Caribbean. It is worth noting that approximately half of all IMGs are from countries with medium or low HDI, countries that may be in significant need of skilled healthcare workers. According to the World Health Organization, 37% of the world’s healthcare professionals are living in North America despite Canada and the United States carrying only 10% of the global disease burden.^31^

These trends in international physician migration give rise to concerns about global health equity. Increasingly, there is a limited supply of healthcare workers in many low- and middle-income countries and the “brain drain” phenomenon has been the subject of exploration in recent studies.^4^,^14^,^32^,^33^ In 2007, the CPSO published a statement on ethical recruitment based on policy papers put forth by the World Health Organization.^34^ Despite this effort at ethical recruitment aimed at discouraging “poaching” from low-income countries, IMGs have the prerogative to choose the country in which they wish to practice medicine and may continue to migrate to higher-income locations in spite of these policies.^3^,^14^ Policy developers must continuously balance physician autonomy with health human resource distribution and global health equity.

Trends in physician migration also underscores policy questions that have been raised about who should make up the physician workforce in Canada.^35^ Given that Canada is becoming an increasingly diverse country, IMGs play an important role in serving the heterogeneous patient population in this country. As we collectively consider this issue, the demographic profile of IMGs is changing as more and more Canadians study abroad with the intention of returning to Canada for post-graduate training and employment. Canadians Studying Abroad (CSAs) now comprise a significant and distinct subset of IMGs in this country. The number of CSAs has grown dramatically since 2000^36^ and an increasing proportion of IMGs who apply for Canadian residency programs each year are CSAs (25% in 2011 compared to 12% in 2008).^37^ It is also becoming increasingly common for CSAs to complete postgraduate training in the United States^38^ and use an alternative licensure route to enter practice in Ontario, highlighting an unanticipated use of the licensure route originally created for American-trained IMG physicians. As the profile of IMGs evolves, we may begin to see even more diverse international migration trends and potentially further use of alternative licensure policies.

### Practice Characteristics

The practice characteristics of physicians in this study differed slightly by licensure route. Of the physicians who were registered through the traditional licensure route between 2000 and 2012, more TLP-IMGs practiced Family Medicine compared to TLP-DMGs. This may be because prior to 2007, IMGs seeking postgraduate placements in Canada could only apply to the second round of residency matches, at which time most specialty (i.e. non-Family Medicine) placements were already secured by DMGs.^13^ ALP-IMGs, on the other hand, had a higher proportion of specialists compared to either TLP-IMGs or -DMGs, possibly due to the increased ability of internationally trained specialists to gain entry through alternative licensure routes. This is reflected in the ALP-Practice Ready IMGs, who either completed their specialist training in an approved jurisdiction or underwent a practice ready assessment in Ontario, as well as in the ALP-North American Trained IMGs.

However, the ALP-Canadian Practice Experience IMGs, who migrated from another Canadian province, had the highest proportion of physicians specializing in Family Medicine than all other groups. This may reflect the fact that some provinces used to issue provisional licenses to IMGs to practice in underserviced areas, but after two years of practice experience, these IMGs gained eligibility to write the College of Family Physicians of Canada (CFPC) exams and would often move elsewhere in Canada. The trend for IMGs to obtain provisional licenses in one province, practice for two years in order to qualify for a full license in Family Medicine, and then migrate to other provinces such as Ontario has been observed previously.^24^ In the future, the use of smaller provinces to access licensure in Ontario will likely subside as licensure requirements become standardized across provinces.

All physicians, regardless of licensure route, practiced predominantly in urban compared to rural locations. Given that increasing physician supply in rural and underserviced areas was a key driver in the development of many health policies, including alternative licensure policies,^11^ this may be evidence of both the ineffectiveness of the current policies and the great need for them. It is possible that some of the ALP-IMGs in this study originally practiced in rural regions through return-of-service agreements but later migrated to urban locations within the province.^23^,^24^,^39^ Urban centers provide more ethnic diversity than rural regions which is preferable for many IMGs,^40^ however this pattern of migration had considerable implications for continued shortages in rural and remote areas and for the continuity of care of patients in those regions.^39^ In 2010, the restrictions on practice locations for return-of-service agreements were reduced, allowing IMGs more choice in where they can begin practicing, and a more comprehensive strategy for northern and rural recruitment of physicians was established.^41^ Research has shown that using IMGs to fill rural needs is not effective,^33^ but physicians coming from a rural background or completing undergraduate or postgraduate training in rural areas are more likely to enter rural practice.^42^ The newly opened Northern Ontario School of Medicine (NOSM) has an explicit goal of physician retention in Northern Ontario and early research shows promise for this self-sufficient model.^43^

### Future Policy Development

Health policies often respond to changing trends in physician demographics and, in turn, the demographic and practice characteristics of physicians influence ongoing policy development. By tracking and reporting on the practice patterns of physicians in Ontario, we aim to contribute to future health human resource planning and facilitate an understanding of the diverse groups of IMGs in the province. This study describes the physicians registered in Ontario between 2000 and 2012, focusing on those who accessed CPSO’s alternative licensure routes. Since 2012, an increasing number of physicians have accessed alternative licensure routes. For example, the number of physicians utilizing the route developed for American-trained physicians has more than doubled in the last three years. Continuing to monitor the characteristics and career trajectories of all IMGs will be necessary to guide effective policy development to be responsive to globalization trends that may impact the medical profession.

One IMG group underrepresented in the literature are the physicians who do *not* successfully obtain licensure. Brain drain from countries with physician shortages often results in brain waste, as many foreign trained doctors are not able to utilize their education and training when they migrate to higher income countries.^14^,^44^–^46^ This phenomenon is due to many factors including lack of coordination between various policy initiatives, lack of knowledge before emigrating about the chances of successfully obtaining licensure, high competition for residency positions, and inability to pass assessments. Although little information currently exists on how many of these unlicensed physicians are in Ontario, one estimate suggests the number may be in the several thousands.^45^ This issue has been recognized by the federal government, which has provided funding for the development of retraining programs for internationally educated health professionals, most of whom are physicians.^47^

Policy development to address physician supply and distribution has always been complex but the evolving nature of globalization compounds these issues. Going forward, policy developers will need to collaborate across systems to carefully consider issues such as global health equity, access to medical education and licensure, ongoing shortages in rural and indigenous communities, and ultimately, the characteristics of the future physician labour force in Ontario. In 2013, the Physician Resource Planning Task Force was developed to coordinate a pan-Canadian health human resources strategy to ensure the appropriate mix, distribution and number of physicians practicing in the country.^48^ A pan-Canadian approach to HHR planning is imperative to ensure policy alignment in light of increasing physician mobility and global complexity.

The ultimate goal of health human resource planning is to ensure that patients receive timely, quality care that is accessible and equitable. In helping to achieve this goal, medical regulators have the challenging role of balancing the provision of access to licensure for physicians while ensuring public protection. The analysis described in the current paper represents the first phase of a comprehensive evaluation focusing on the CPSO’s registration policies and licensure routes. Using the current data for this cohort of IMGs as a starting point, future studies will seek to assess the quality of medical practice of these physicians. Such lines of research may shed light on how regulation can improve integration and education for doctors already in the system and the future resource of internationally trained physicians. The medical regulator is but one player in a complex system attempting to address health human resource challenges in an increasingly globalized landscape. Developing effective strategies to address the above issues will not be easy and will rely on sound research, a willingness to address difficult questions, and a tolerance for the constantly evolving nature of globalized medical education and practice.

## Figures and Tables

**Table 1:** Description of the Traditionally Licensed Physicians and the Alternatively Licensed IMGs

Physician Group	Physician Sub-Groups	Description	Undergrad Location	Postgrad Location
**Traditionally Licensed Physicians (TLP)**	**DMG**	Physicians who completed undergraduate medical education in North America (Canada and the US), post-graduate training in Canada, and passed the Canadian licensing examinations.	North America	Canada
	**IMG**	Physicians who completed undergraduate medical education abroad, post-graduate training in Canada, and passed the Canadian licensing examinations.	Abroad	Canada
**Alternatively Licensed IMGs (ALP-IMG)**	**Canadian Practice Experience IMG**	Internationally trained physicians who gained eligibility to practice in Ontario by practising in another Canadian province. *Includes IMGs who utilized either AIT legislation or who were eligible to write CFPC exams route through a minimum of two years of Canadian practice experience.*	Abroad	Abroad
	**Practice Ready IMG**	Internationally trained physicians who were deemed eligible to practice in Ontario without completing additional Canadian training or practicing in another Canadian province. *Includes IMGs who completed training in a RCPSC-approved jurisdiction or underwent a practice ready assessment (APIMG) in Ontario and were deemed practice ready.*	Abroad	Abroad
	**North American Trained IMG**	Internationally trained physicians who gained eligibility to practice in Ontario by completing postgraduate training in North America (Canada and the US). *Includes IMGs who completed postgraduate training exclusively in North America and those who completed postgraduate training abroad and completed additional North American training.*	Abroad	North America

DMG – Domestic Medical Graduate || IMG – International Medical Graduate || Agreement on Internal Trade (AIT) – labour mobility legislation that allows free movement of physicians across provinces || APIMG – Assessment Program for International Medical Graduates|| CFPC – College of Family Physicians of Canada RCPSC – Royal College of Physicians and Surgeons of Canada

**Table 2:** Demographic and practice characteristics of physicians registered from 2000 to 2012

	Traditionally Licensed Physicians (TLP)			Alternatively Licensed IMGs (ALP-IMGs)			
	Total	DMG	IMG	Total	Canadian Practice Experience IMG	Practice Ready IMG	North American Trained IMG
**Total (n)**	**10,595**	**7,762**	**2,833**	**655**	**132**	**146**	**377**
**Age at registration, mean ± SD**	31.1 ± 6.5	28.9 ± 4.5	36.9 ± 7.4	39.6 ± 7.8	44.2 ±10.5	39.2 ±6.1	38.1 ±6.6
**Sex, % male (n)**	49% (5203)	48% (3684)	54% (1519)	59% (387)	73% (96)	55% (80)	56% (211)
**Region of medical school, % (n)**							
Canada	72% (7633)	98% (7633)	-	-	-	-	-
United States	1% (129)	2% (129)	-	-	-	-	-
Middle East	6% (680)	-	24% (680)	17% (111)	23% (30)	15% (22)	16% (59)
Caribbean	2% (258)	-	9% (258)	13% (88)	9% (12)	-	19% (71)
Indian Subcontinent	6% (621)	-	22% (621)	28% (185)	24% (31)	23% (33)	32% (121)
Africa (excl. South Africa)	2% (237)	-	8% (237)	9% (60)	14% (19)	7% (10)	8% (31)
South Africa	1% (114)	-	4% (114)	6% (38)	10% (13)	17% (25)	-
Eastern Europe	3% (362)	-	13% (362)	12% (78)	8% (10)	6% (9)	16% (59)
Western Europe	3% (315)	-	11% (315)	6% (41)	9% (12)	12% (17)	3% (12)
Australia & New Zealand	1% (76)	-	3% (76)	1% (7)	-	-	-
South & Central America	1% (75)	-	3% (75)	4% (27)	-	12% (17)	2% (8)
Asia (South & East)	1% (95)	-	3% (95)	3% (20)	-	-	4% (15)
**HDI of medical school country, % (n)**							
Very high human development	79% (8404)	100%	23% (642)	13% (83)	15% (20)	16% (24)	10% (39)
High human development	9% (974)	-	34% (974)	35% (230)	21% (28)	30% (44)	42% (158)
Medium human development	8% (883)	-	31% (883)	37% (241)	46% (60)	44% (64)	31% (117)
Low human development	3% (334)	-	12% (334)	15% (101)	18% (24)	10% (14)	17% (63)
**Specialty/Practice Focus, % (n)**							
Family Medicine	49% (5264)	47% (3641)	57% (1623)	44% (287)	67% (89)	32% (47)	40% (151)
Medical Specialties	18% (1916)	19% (1484)	15% (432)	17% (114)	8% (11)	16% (23)	21% (80)
Surgical Specialties	13% (1342)	14% (1093)	9% (249)	9% (57)	-	21% (30)	6% (21)
Diagnostic Specialties	6% (600)	5% (422)	6% (178)	6% (37)	6% (8)	-	7% (27)
Psychiatry	5% (527)	5% (394)	5% (133)	9% (56)	7% (9)	-	11% (40)
Pediatrics	4% (432)	4% (335)	3% (97)	8% (53)	-	10% (15)	9% (35)
Anesthesiology & Critical Care	5% (514)	5% (393)	4% (121)	8% (51)	5% (6)	15% (22)	6% (23)
**Practice Location % (n)**	**9,536**	**7,030**	**2,506**	**586**	**100**	**141**	**345**
Urban	85% (8086)	85% (5984)	84% (2102)	78% (457)	78% (78)	72% (101)	81% (278)
Suburban	11% (1035)	10% (702)	13% (333)	18% (107)	15% (15)	24% (34)	17% (58)
Rural	4% (415)	5% (344)	3% (71)	4% (22)	7% (7)	4% (6)	3% (9)

DMG – Domestic Medical Graduate || IMG – International Medical Graduate || ± SD or prevalence (%) and n || - represents values that are not applicable or supressed due to small cell sizes and/or to maintain privacy ||
